# Immortalized myogenic cells from congenital muscular dystrophy type1A patients recapitulate aberrant caspase activation in pathogenesis: a new tool for MDC1A research

**DOI:** 10.1186/2044-5040-3-28

**Published:** 2013-12-06

**Authors:** Soonsang Yoon, Guido Stadler, Mary Lou Beermann, Eric V Schmidt, James A Windelborn, Peter Schneiderat, Woodring E Wright, Jeffrey Boone Miller

**Affiliations:** 1 Departments of Neurology and Physiology & Biophysics, Neuromuscular Biology & Disease Group, Boston University School of Medicine, Boston, MA, USA; 2 Department of Cell Biology, University of Texas Southwestern Medical Center at Dallas, Dallas, TX, USA; 3 Wellstone Program and Department of Cell & Developmental Biology, University of Massachusetts School of Medicine, Worcester, MA USA; 4 Friedrich-Baur-Institute, Department of Neurology, Ludwig-Maximilians-University of Munich, Munich, Germany

**Keywords:** Caspase-3 activation, Congenital muscular dystrophy, Immortalization of myogenic cells, Laminin-α2-deficiency, Myotube, Telomerase

## Abstract

**Background:**

Congenital muscular dystrophy Type 1A (MDC1A) is a severe, recessive disease of childhood onset that is caused by mutations in the *LAMA2* gene encoding laminin-α2. Studies with both mouse models and primary cultures of human MDC1A myogenic cells suggest that aberrant activation of cell death is a significant contributor to pathogenesis in laminin-α2-deficiency.

**Methods:**

To overcome the limited population doublings of primary cultures, we generated immortalized, clonal lines of human MDC1A myogenic cells via overexpression of both CDK4 and the telomerase catalytic component (human telomerase reverse transcriptase (hTERT)).

**Results:**

The immortalized MDC1A myogenic cells proliferated indefinitely when cultured at low density in high serum growth medium, but retained the capacity to form multinucleate myotubes and express muscle-specific proteins when switched to low serum medium. When cultured in the absence of laminin, myotubes formed from immortalized MDC1A myoblasts, but not those formed from immortalized healthy or disease control human myoblasts, showed significantly increased activation of caspase-3. This pattern of aberrant caspase-3 activation in the immortalized cultures was similar to that found previously in primary MDC1A cultures and laminin-α2-deficient mice.

**Conclusions:**

Immortalized MDC1A myogenic cells provide a new resource for studies of pathogenetic mechanisms and for screening possible therapeutic approaches in laminin-α2-deficiency.

## Background

Congenital muscular dystrophy Type1A (MDC1A) is an autosomal recessive disease caused by mutations in the *LAMA2* gene that encodes the extracellular protein laminin-α2 [1]. Mutations that result in complete loss of laminin-α2 function result in severe neuromuscular dysfunction, whereas mutations that result in partial loss of function are associated with less severe disease [2]. In skeletal muscles, laminin-α2 assembles with laminin-β1 and -γ1 to form laminin-211. Heterotrimeric laminins that include laminin-α2 have been termed merosins, and MDC1A has thus also been known as merosin-deficient congenital muscular dystrophy. Laminin-α2 has multiple binding partners in both the extracellular matrix and on the plasma membrane [3] so that loss of laminin-α2 is accompanied by both structural deficits and aberrant cell signaling.

Primary cultures of myogenic cells from human MDC1A patients have proven useful for analyzing molecular mechanisms of MDC1A pathogenesis in skeletal muscle. For example, myotubes formed in primary cultures of human MDC1A myoblasts in the absence of exogenous laminin show both a several-fold increase in caspase-3 activity and increased cell death compared to myotubes formed from healthy control myoblasts [4]. The increased caspase-3 activity in MDC1A myotubes *in vitro* appears to recapitulate the similarly increased caspase-3 activity seen in the skeletal muscles of laminin-α2-deficient mice and human MDC1A patients *in vivo*[5-9]. Thus, aberrant activation of caspase enzymatic activity is a cell autonomous property of laminin-α2-deficient myotubes. The aberrant caspase activation and cell death in muscle cells of MDC1A model systems is mediated by a BAX/KU70-dependent signaling pathway [4]. Importantly, inhibition of aberrant cell death in the skeletal muscles of laminin-α2-deficient mice leads to a significant amelioration of pathology, including a several-fold increase in lifespan and improved motor behavior [4,10,11], thereby demonstrating that aberrantly increased cell death is both a significant contributor to the overall pathology and a potential therapeutic target in human MDC1A.

The use of primary cultures of human MDC1A myogenic cells to analyze pathogenetic mechanisms has been constrained both by the small number of donors and by the limited replication capacity (typically approximately 50 to 60 population doublings) of human myogenic cells in primary culture. However, the replication limits of human myogenic cells can be overcome through forced expression of CDK4 and hTERT [12-14]. Using this technique, we now report the preparation and analysis of immortalized, clonal lines of human MDC1A myogenic cells. We found that the immortalized cells not only retained the capacity to differentiate into myotubes but also showed the aberrant activation of caspase activity as seen in primary cultures. This is the first report of immortalized human myogenic cells that recapitulate such a marked pathological change. Thus, these immortalized MDC1A myogenic cells can provide an essentially unlimited number of cells for study of MDC1A pathogenetic mechanisms, as well as for the identification and *in vitro* validation of therapeutic targets and strategies, including by high-throughput screening.

## Methods

### Immortalization and cell cloning

Immortalization of myoblasts and isolation of myogenic clones was performed as previously described [12-14]. In brief, mouse CDK4 and hTERT cDNAs were inserted into pBabe vectors containing neomycin- and hygromycin-resistance genes, respectively. LoxP sites were included in the hTERT vector to allow optional excision of the hTERT expression cassette by Cre recombinase. To produce retroviral vectors, these plasmids were transfected into the Phoenix ecotropic packaging cell and the virus-containing supernatant was used to infect the amphotropic packaging cell line PA317 [15] to obtain stable virus-producing cell lines after selection with 0.5 mg/mL G418 or hygromycin (EMD Biosciences, San Diego, CA, USA). Infections were done with 2 μg/mL polybrene (Sigma-Aldrich). Clonal colonies were grown from the immortalized population by limiting dilution culture, and clonally-related cells were analyzed for CD56 expression by flow cytometry and for fusion potential in differentiation medium. Several independent clonal lines were isolated from each immortalized population and expanded for further assays. Telomere length and telomerase activity were assayed as before [13,16].

### Human myogenic cells

Table 1 summarizes the human myogenic cells used in this study. All human cells were obtained from German or USA biobanks (Table 1 and described below). All cells were anonymized prior to receipt and no personal identifications were available to us. The cells had been produced prior to our study from muscle biopsies collected under protocols approved by the appropriate institution that included informed donor consent and approval to publish results in accordance with standards of the Helsinki Declaration [17,18]. Because our studies were of human cells that were obtained from cell banks and for which personal identification data were not obtainable by us, the studies were classified as exempt from Human Studies review by the Boston University Institutional Review Board in accordance with USA Department of Health and Human Services policy (http://www.hhs.gov/ohrp/humansubjects/guidance/45cfr46.html#46.101, accessed November, 4, 2013).

**Table 1:** **Primary and CDK4** + **hTERT immortalized myogenic cells used in this study**

**Disease status**	**Donor, age**	**Source**	**Type**	**Name**
MDC1A	Male, 4 months	MTCC Munich	Primary	38/03
		This work	Immortalized	38/03-ct4
MDC1A	Male, 8 months	MTCC Munich	Primary	96/04
		This work	Immortalized	96/04-ct8
FSHD	Male, 67 years	Wellstone	Primary	15Abic
		Ref 16	Immortalized	15Abic-ct24
Healthy control	Female, 60 years	Wellstone	Primary	15Vbic
		Ref 16	Immortalized	15Vbic-ct16
Healthy control	Male, 36 years	MTCC Munich	Primary	2/08
		This work	Immortalized	2/08-ct7

Primary MDC1A myoblasts from two different patients, designated as strains 38/03 and 96/04, were provided by the Muscle Tissue Culture Collection (MTCC) at the University of Munich (http://www.baur-institut.de/forschung/muskelbank/, accessed November 4, 2013) and were obtained from 4-month-old and 8-month-old male donors, respectively. Each donor had a clinical diagnosis of MDC1A with absence of laminin-α2 [4]. As controls, we analyzed primary myoblasts of a healthy 36-year-old man (unpublished strain 2/08, provided by the MTCC), as well as myoblasts derived from a biceps biopsy of a healthy 60-year-old woman, termed 15Vbic [17,18]. As a disease control, we analyzed myoblasts derived from a biceps biopsy of a 67-year-old man with facioscapulohumeral dystrophy (FSHD), termed 15Abic [16-18]. Primary 15Abic and 15Vbic cells were prepared by and obtained from the Sen. Paul D. Wellstone Cooperative Research Center for FSHD (http://www.umassmed.edu/wellstone/materials.aspx, accessed November 4, 2013) and immortalization of these 15Abic and 15Vbic cells was reported previously [16]. Due to the young age of the MDC1A donor, it was not possible to obtain control myoblasts from age-matched donors. After immortalization, each clonal line was given a new identifier consisting of the original name followed by ‘-ct’ (for CDK4 + hTERT) and a clone number. Thus, 38/03-ct4 was the fourth clonal line derived from CDK4/hTERT immortalized 38/03 cells. Requests for immortalized 38/03-ct4, 96/04-ct8, and 2/08-ct7 myoblasts (Table 1) should be addressed to Dr. Peter Schneiderat (Peter.Schneiderat@med.uni-muenchen.de); and requests for immortalized 15Abic and 15Vbic myoblasts should be addressed to the Director of the Wellstone FSHD Center (charles.emersonjr@umassmed.edu).

### Cell culture

Cells were cultured on Lab-Tek Permanox chamber slides (Nalge Nunc, Rochester, NY, USA) coated with 40 μg/mL poly-D-Lysine or 1% gelatin. In some cases as noted, slides were coated at 2 μg/cm^2^ with human placental laminin (Sigma-Aldrich cat. #L6274). Proliferating myoblasts were cultured at subconfluence in a high serum growth medium and differentiation was induced as cells neared confluence by switching the cultures to a low serum differentiation medium as described [17,18]. Where noted, Laminin-111 (Sigma-Aldrich cat. #L2020 or BD Bioscience cat. #354239) was added to the culture medium at 5 μM. Cells were cultured under 5% CO_2_ at ambient oxygen concentration (normoxia), except, in some cases as noted, when cells were cultured under a low oxygen atmosphere of 2% O_2_, 5% CO_2_, 93% N_2_ (hypoxia) in gas-tight chambers as described [19].

### Caspase enzyme assays

Caspase enzymatic activity was measured in cell homogenates using either the CaspACE Colorimetric Caspase-3 Activity Assay (50 to 100 μg protein per assay; Promega, Madison, WI, USA) or the more sensitive luminescence-based Caspase-Glo 3/7 Assay System (5 μg protein per assay; Promega) according to the manufacturer’s instructions and with signal detection on a Safire2 or Infinite M1000 microplate reader (Tecan, Durham, NC, USA).

### Antibodies and immunocytochemistry

Myosin heavy chain isoforms were detected with one of three antibodies: (1) mouse mAb F59 [20] used at 1:10 dilution of hybridoma supernatant; (2) mouse mAb F20 [21] (used at 1:10; Developmental Studies Hybridoma Bank, Iowa City, IA, USA), or (3) rabbit anti-MYH3 (Sigma-Aldrich, St. Louis, MO, USA). Desmin was detected with mouse mAb D1033 (Sigma-Aldrich) used at 1:100 for 1 h. Activated caspase-3 antibody was from Cell Signaling Technologies (Beverly, MA, USA; cat. #9661, used at 1:400); and KU70 antibody was from Novus Biologicals (Littleton, CO, USA; cat #NB100-1915, used at 1:300). Dr. Lydia Sorokin (University of Münster) generously provided the rat anti-laminin-α2 mAb 4H8-2 which reacts with an epitope within the L4b globular domain [22]. Cultures were fixed with 4% formaldehyde or 100% methanol. Primary antibody binding was visualized with appropriate species-specific secondary antibodies conjugated to either Alexa Fluor 488 or Alexa Fluor 594 (Life Technologies, Grand Island, NY, USA). Slides were imaged using a Nikon E800 microscope (Melville, NY, USA) with SPOT Software (version 4.1) and SPOT Insight camera (Diagnostic Instruments, Sterling Heights, MI, USA).

## Results and discussion

**Figure 1:**
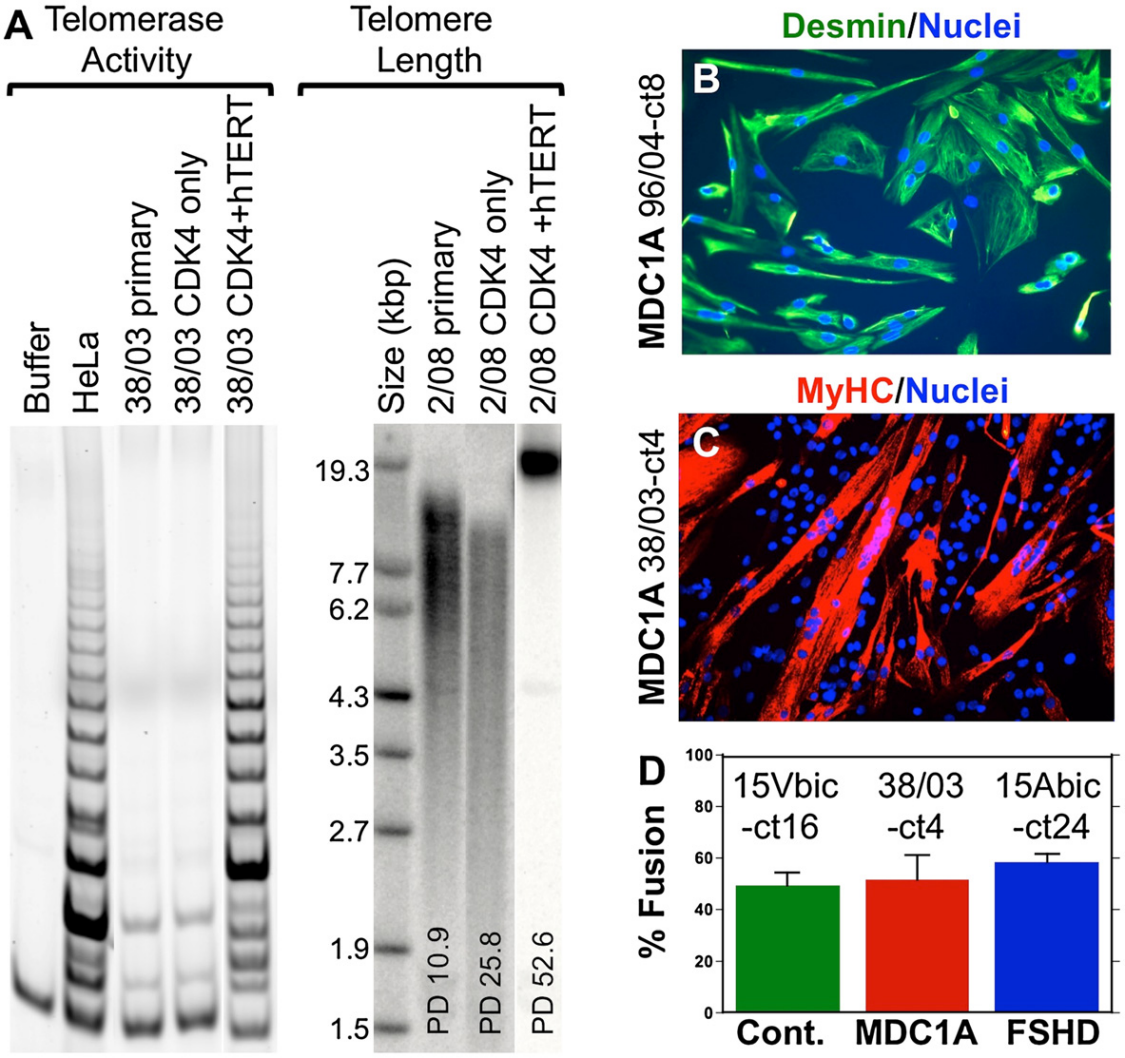
**Characterization of immortalized compared to primary myogenic cells. (A)** CDK4 + hTERT immortalized cells had higher telomerase enzymatic activity (left) and maintained longer telomeres (right) than primary cells or cells with CDK4 only. Results from 38/03 MDC1A cells (telomerase activity by telomeric repeat amplification protocol assay) and healthy control 2/08 cells (telomere length by hybridization assay) are shown as examples. The cervical carcinoma cell line HeLa served as a positive control. Lanes re-arranged for presentation. PD = population doublings. **(B)** 100% of CDK4 + hTERT immortalized cells expressed desmin (green), with MDC1A 96/04-ct8 cells shown as an example. **(C)** CDK4 + hTERT immortalized MDC1A myogenic cells showed normal differentiation by fusing into multinucleate cells that expressed myosin heavy chain (MyHC, red) isoforms. **(D)** Similar percentages of nuclei were incorporated into multinucleate (≥2 nuclei) myotubes formed from immortalized healthy control, MDC1A, and FSHD myoblasts. Error bars = SD, *n* = 5.

Using forced expression of CDK4 and hTERT followed by cell cloning, we first produced immortalized myogenic cell lines from primary human myoblasts obtained from MDC1A (38/03, 96/04), normal control (2/08, 15Vbic), and FSHD (15Abic) donors (Table 1 and Figure 1). The FSHD cells served as a disease control to determine if pathological changes were disease-specific or shared. Though primary myoblast cultures reached a replicative limit at approximately 50 to 60 cumulative population doublings, the immortalized cells proliferated indefinitely (not shown, compare to [13]). Cells that were CDK4 plus hTERT immortalized had higher telomerase enzymatic activity and maintained longer telomeres at higher population doublings than either primary cells or cells with CDK4 alone (Figure 1A). Culture under low oxygen conditions (2% O_2_, 5% CO_2_, 93% N_2_) did not significantly alter proliferation or differentiation of the immortalized normal, MDC1A, or FSHD lines compared to culture under normoxic conditions (not shown).

**Figure 2:**
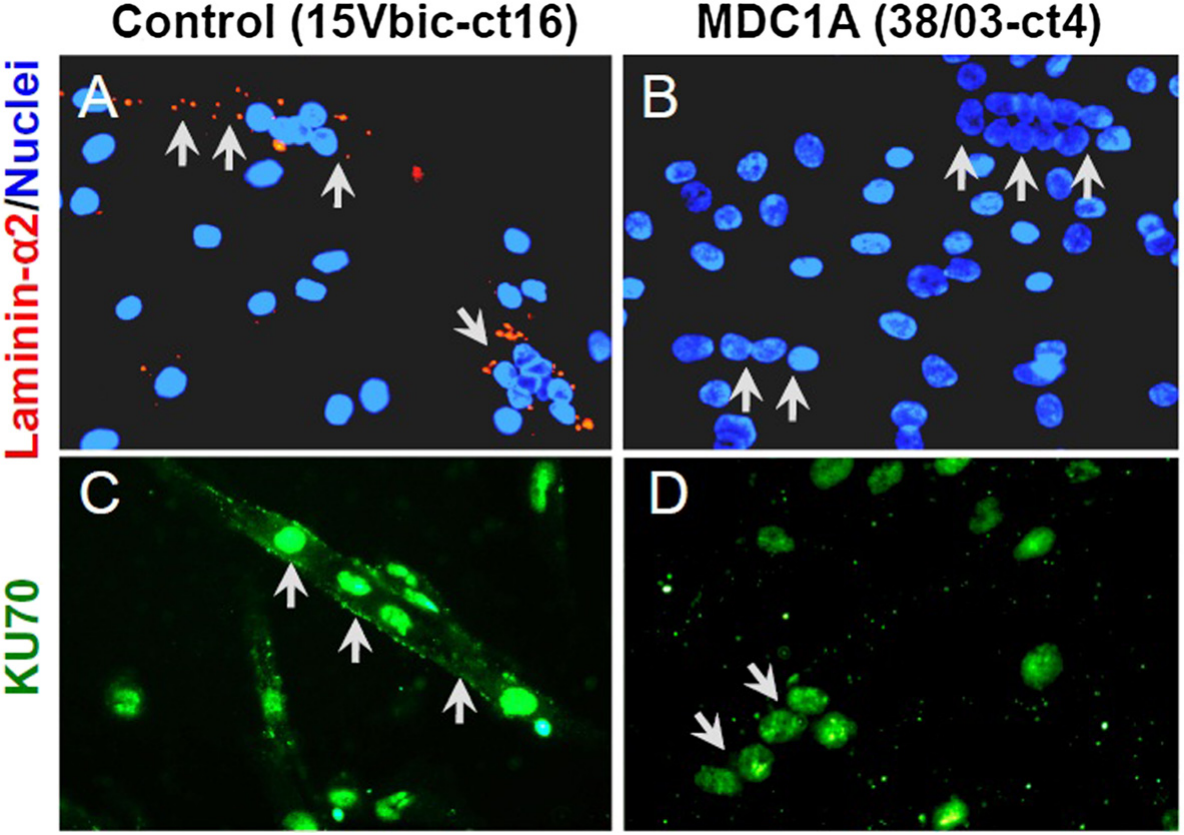
**Immortalized MDC1A myogenic cells did not express laminin-α2 and showed an altered distribution of KU70. (A)** Laminin-α2 (red) appeared in a punctate pattern in multinucleate myotubes (arrows) formed from immortalized healthy control myoblasts. **(B)** As expected, no laminin-α2 was found in myotubes (arrows) formed from immortalized MDC1A myoblasts. **(C)** Immortalized myogenic cells from healthy control donors showed KU70 (green) both in the cytoplasm (arrows) and in nuclei. **(D)** KU70 (green) was restricted to nuclei of immortalized MDC1A myogenic cells.

The clonal, immortalized myogenic cells were 100% positive for expression of the muscle-specific intermediate filament protein desmin (Figure 1B), whereas primary cultures were 70% to 95% desmin-positive (not shown), as was consistent with a small proportion of non-myogenic cells in the non-clonal primary cultures. Immortalized MDC1A, FSHD, and normal control myogenic cells all formed multinucleate myotubes when switched to low serum differentiation medium, and, as in primary cell cultures, the percentage of nuclei that were within multinucleate cells was similar for disease and control cultures (Figure 1C, D, and not shown). We also confirmed that myotubes formed from immortalized MDC1A myoblasts failed to express laminin-α2, whereas myotubes formed from immortalized control myoblasts did express laminin-α2 (Figure 2A, B), thus demonstrating that the laminin-α2-deficient phenotype was maintained in the immortalized MDC1A cultures.

We next examined whether immortalized MDC1A myogenic cells also showed the pathological changes that we had previously found in primary MDC1A cultures [4]. We first compared KU70 immunostaining patterns in cultures of immortalized control and MDC1A myogenic cells. We found that KU70 immunostaining was restricted to the nuclei of immortalized MDC1A myogenic cells, whereas both the cytoplasm and nuclei of immortalized normal control cells showed KU70 staining (Figure 2A, B). Because primary cultures of MDC1A myogenic cells also show decreased KU70 expression in the cytoplasm [4], it is clear that immortalization did not affect this aberrant property of MDC1A myogenic cells. KU70 is a multifunctional protein with roles in the nucleus, cytoplasm, and perhaps at the cell surface [23]. In the cytoplasm, KU70 normally binds to BAX, thereby inhibiting BAX activation and subsequent cell death [4,24-26]. Loss of KU70 from the cytoplasm would promote BAX activation and cell death, which is consistent with the increased cell death phenotype in laminin-α2-deficient mouse muscles and MDC1A human muscles [5-9].

**Figure 3:**
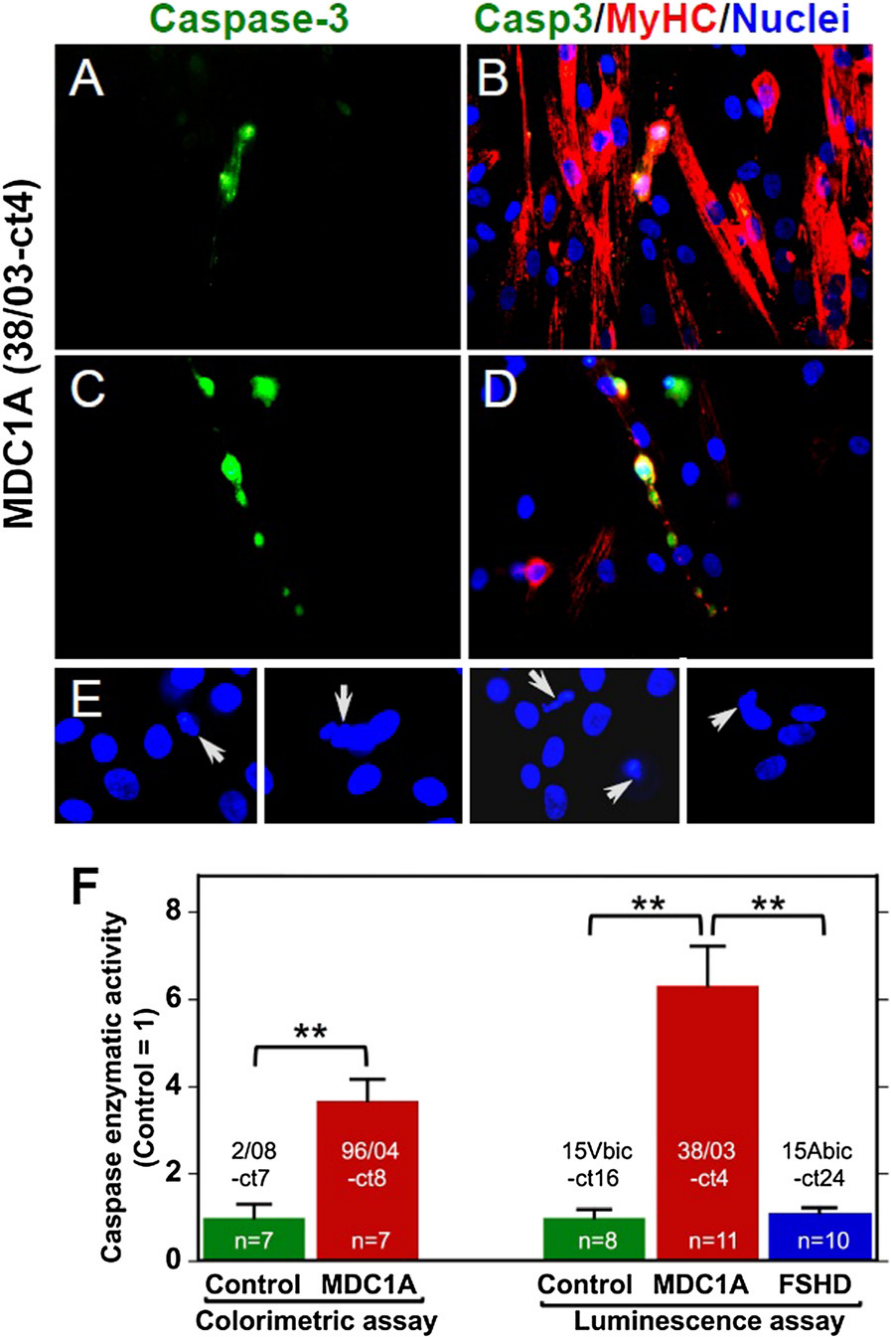
**Caspase**-**3 activity was aberrantly activated in myotubes formed from immortalized MDC1A myoblasts. (A, B, C, D)** Activated caspase-3 (green) was found in approximately 1% to 3% of the MyHC-expressing (red) differentiated cells in cultures of immortalized MDC1A cells. Two examples of 38/03-ct4 cultures are shown after 4 days in differentiation medium. **(E)** Arrows indicate nuclei with aberrant morphology that were in caspase-3-positive cells (caspase staining not shown). Nearby nuclei in caspase-3-negative cells showed normal morphology. Four fields are shown. **(F)** Quantification of caspase-3 enzymatic activity by two different assays showed that, after 4 days in differentiation medium, cultures of immortalized MDC1A cells (96/04-ct8 and 38/03-ct4) had significantly elevated levels of caspase-3 compared to cultures of immortalized healthy control cells (2/08-ct and 15Vbic-ct16) or immortalized FSHD (15Abic-ct24) cells. **P <001 (*t*-test, ‘n’ as shown).

Our next step was to examine caspase-3 activation in cultures of immortalized MDC1A *vs*. immortalized normal and FSHD myogenic cells. Caspase-3 activation is associated with activation of the BAX-mediated pathway of cell death in MDC1A cell cultures [4]. Using immunohistochemistry with an antibody specific for the cleaved, enzymatically active form of caspase-3, we found positive immunostaining in approximately 1% to 3% of the differentiated, myosin heavy chain-positive (MyHC) cells in MDC1A cultures (Figure 3A to D). For example, in one survey of an MDC1A 38/03-ct4 culture after 4 days of differentiation on gelatin, we found caspase-3 immunostaining in 15 out of 1,084 (1.4%) of the MyHC-positive cells in the culture. The caspase-3 immunostaining in MDC1A cells appeared to often fill the cytoplasm and was sometimes also in nuclei as expected for ongoing cell death (Figure 3A to D). In some cases, the caspase-3-positive cells appeared to be undergoing degeneration as evidenced by fragmented and/or aggregated MyHC staining (Figure 3C, D). Furthermore, nuclei in caspase-3-positive cells were often irregularly shaped, condensed, or fragmented (Figure 3E), which are signs of incipient cell death. We did not see such caspase-3-positive cells with aberrant nuclei in differentiated cultures of immortalized normal or FSHD myogenic cells (not shown).

The finding that only a small fraction of the differentiated, myosin-expressing cells were positive for caspase-3 at any one time suggests that onset of cell death was asynchronous in the differentiating MDC1A cultures. Caspase-3-positive cells typically have a short half-life (<5 h); eventually detach from the culture dish [4]; and could possibly be replaced by remaining undifferentiated myoblasts in the cultures. The mechanism(s) that underlie the progression of cells from a state in which there are limited signs of pathology (for example, KU70 reduced in the cytoplasm) to a state with high level activation of caspase-3 followed by cell death remain to be determined in future work.

Finally, to confirm the immunocytochemistry results, we measured caspase-3 enzymatic activity in differentiated cultures of MDC1A *vs*. normal and FSHD myogenic cells. After 4 days of differentiation, cultures of MDC1A cells had significantly more caspase-3 enzymatic activity than did cultures of normal control or FSHD cells (Figure 3F). This approximate four- to six-fold increase in caspase-3 activity in immortalized MDC1A lines was similar to the increase we saw previously in primary MDC1A *vs*. primary normal cultures [4]. We found similar results with two different immortalized MDC1A lines (38/03-ct4 and 96/04-ct8) and with two different caspase-3 enzymatic activity assays. Culture under low oxygen did not alter the extent of caspase-3 activation (not shown). The increased caspase-3 activation in the MDC1A cultures was at least partially laminin-dependent, as growth on human placental laminin (which includes laminin-211) or in the presence of mouse laminin-111 was sufficient to prevent approximately 30% to 50% of the aberrant increase in caspase-3 (not shown), a result similar to that we found previously in primary MDC1A cultures [4].

In summary, we have immortalized laminin-α2-deficient MDC1A myogenic cells and shown that the immortalized cells not only retain the capacity for differentiation, but also recapitulate cell autonomous pathological changes that have been reported in primary MDC1A myogenic cell cultures, in laminin-α2-deficient mouse muscles, and in human MDC1A muscles [4-9]. Among these changes were a reduction in the amount of KU70 in the cytoplasm and aberrant activation of caspase-3 with associated abnormalities of nuclear morphology. The immortalized MDC1A myogenic cells should provide an essentially unlimited source of laminin-α2-deficient cells for future studies. In particular, these cells will be valuable for studies of myogenic cell-autonomous mechanisms in MDC1A pathology, including, for example, aberrant induction of cell death and increased autophagy [4,27]. Combining results from studies of the human MDC1A myogenic cells with results from studies of laminin-α2-deficient mice should be particularly useful for further analyses of disease mechanisms. Pathological changes that arise due to interactions of human MDC1A myogenic cells with motor nerve, vascular, inflammatory, or connective tissue cells could possibly be studied in co-cultures. Xenotransplant models might also be useful if the immortalized MDC1A myogenic cells can form a significant number of innervated myofibers after transplant into immunodeficient mice [14]. Finally, the immortalized MDC1A cells and the pathological changes in these cells that we have identified should be useful for developing cell-based screening assays, including high-throughput screening, as part of pre-clinical studies to identify therapeutic interventions that reverse MDC1A pathology.

## Conclusions

Immortalized myogenic cells from laminin-α2-deficient MDC1A patients recapitulate aspects of MDC1A pathology including aberrant induction of caspase-3 and KU70 relocalization. The immortalized MDC1A cells provide a new resource for studies of pathogenetic mechanisms and for screening possible therapeutic approaches in laminin-α2-deficiency.

## Competing interests

The authors declare that they have no competing interests.

## Authors’ contributions

GS and WEW developed the immortalization method, immortalized and cloned the normal, MDC1A, and FSHD lines, and assayed telomerase activity and telomere length (Figure 3A). SY, MLB, EVS, and JAW carried out the remaining experimental studies (Figures 1B-D, 2, and 3, and data in text) which were planned with JBM. PS provided primary MDC1A and control myogenic cells and biobanking services. JBM wrote the manuscript. All authors participated in editing the manuscript. All authors read and approved the final manuscript.
